# Neo-sex Chromosomes in the Monarch Butterfly, *Danaus plexippus*

**DOI:** 10.1534/g3.117.300187

**Published:** 2017-08-23

**Authors:** Andrew J. Mongue, Petr Nguyen, Anna Voleníková, James R. Walters

**Affiliations:** * Department of Ecology and Evolutionary Biology, University of Kansas, Lawrence, Kansas 66046; † Faculty of Science, University of South Bohemia in České Budéjovice, 370 05 České Budějovice, Czech Republic; ‡ Institute of Entomology, Biology Centre of the Czech Academy of Sciences, Czech Academy of Sciences, 370 05 České Budějovice, Czech Republic

**Keywords:** sex chromosomes, evolution, Lepidoptera, genomics, chromosomal fusion

## Abstract

We report the discovery of a neo-sex chromosome in the monarch butterfly, *Danaus plexippus*, and several of its close relatives. Z-linked scaffolds in the *D. plexippus* genome assembly were identified via sex-specific differences in Illumina sequencing coverage. Additionally, a majority of the *D. plexippus* genome assembly was assigned to chromosomes based on counts of one-to-one orthologs relative to the butterfly *Melitaea cinxia* (with replication using two other lepidopteran species), in which genome scaffolds have been mapped to linkage groups. Sequencing coverage-based assessments of Z linkage combined with homology-based chromosomal assignments provided strong evidence for a Z-autosome fusion in the *Danaus* lineage, involving the autosome homologous to chromosome 21 in *M. cinxia*. Coverage analysis also identified three notable assembly errors resulting in chimeric Z-autosome scaffolds. Cytogenetic analysis further revealed a large W chromosome that is partially euchromatic, consistent with being a neo-W chromosome. The discovery of a neo-Z and the provisional assignment of chromosome linkage for >90% of *D. plexippus* genes lays the foundation for novel insights concerning sex chromosome evolution in this female-heterogametic model species for functional and evolutionary genomics.

Major rearrangements of karyotype and chromosome structure often have substantial evolutionary impacts on both the organisms carrying such mutations and the genes linked to such genomic reorganization (Lynch and Walsh 2007; Soltis and Soltis 2012). Additionally, such large-scale chromosomal mutations often present novel opportunities to investigate molecular, evolutionary, and functional genetic processes, for instance the evolution of neo-sex chromosomes, which can arise from the fusion of an autosome with an existing and well-differentiated allosome. This effectively instantaneous transformation of a formerly autosomal set of genes into sex-linked loci is fertile ground for advancing our understanding of the distinct set of evolutionary forces acting on sex chromosomes relative to autosomes (Bachtrog *et al.* 2009; Pala *et al.* 2012a; Bachtrog 2013; Šíchová *et al.* 2013). Furthermore, when such an event is observed in a tractable genetic model system, there is opportunity to explore the functional and mechanistic changes associated with sex chromosome evolution. The congruence of neo-sex chromosomes existing in a model system is relatively rare, although there are some notable examples. For example, independent origins of neo-sex chromosomes are known in *Drosophila* fruit flies (Counterman *et al.* 2004; Flores *et al.* 2008; Bachtrog *et al.* 2009; Zhou *et al.* 2013; Brown and Bachtrog 2014; Nozawa *et al.* 2014) and stickleback fish, where neo-sex chromosomes appear to play an important role in reproductive isolation between incipient species (Kitano *et al.* 2009; Yoshida *et al.* 2014; White *et al.* 2015).

Looking beyond these established model systems, the rapid expansion of genomic technologies has allowed extensive analyses of gene content, sex-biased gene expression, dosage compensation, and sequence divergence for recently evolved sex chromosomes among a very diverse set of organisms including several insect lineages [teleopsid flies, a grasshopper, and Strepsiptera (Baker and Wilkinson 2010; Mahajan and Bachtrog 2015; Palacios-Gimenez *et al.* 2015)], vertebrates [mammals and birds (Zhou *et al.* 2008; Pala *et al.* 2012a; Murata *et al.* 2015)], and plants [*Silene* and *Rumex* genera (Hough *et al.* 2014; Charlesworth 2015; Papadopulos *et al.* 2015)]. A clear consensus emerges from this research that the lack of recombination associated with sex chromosomes catalyzes a cascade of evolutionary changes involving the degeneration of one allosome, the accumulation of genes with sex-biased expression, increased evolutionary rates, and often, but not always, the acquisition of dosage compensation. Yet many of the details in this process remain elusive and unresolved, including the rate of allosome divergence, the role of positive selection *vs.* drift, the importance of sex-specific selection, and the mechanisms underlying dosage compensation or the reasons for its absence. It is therefore important to continue identifying new opportunities for novel insight into the evolution of sex chromosomes.

Overwhelmingly, research on sex chromosomes occurs in male-heterogametic (XY) species (Vicoso and Charlesworth 2006; Ellegren 2011; Bachtrog 2013; Parsch and Ellegren 2013). This is particularly true for neo-sex chromosomes, where contemporary genomic analyses of neo-Z or neo-W chromosomes are currently lacking, with one notable exception for birds (Pala *et al.* 2012a). This imbalance is unfortunate, because WZ sex determination offers the novel framework of female-specific selection during the evolution of heterogamety and is a common form of sex determination in both vertebrates and invertebrates. Birds are the most prominent vertebrate taxon that is female heterogametic, but it appears that avian neo-sex chromosomes are quite rare and indeed absent from prominent model species, *e.g.*, chicken, zebra finch (Nanda *et al.* 2008; Pala *et al.* 2012b). Fishes and squamates seem to be far more labile in sex chromosome constitution, with numerous independent transitions between male and female heterogamety and relatively frequent sex–autosome fusions (Pennell *et al.* 2015), thus there are potentially great opportunities in these taxa. However, no tractable WZ model system with neo-sex chromosomes has been identified for these lineages.

For many reasons, Lepidoptera (moths and butterflies) may be the most promising female-heterogametic taxon for studying neo-sex chromosomes. Synteny, *i.e.*, the chromosomal placement of orthologous genes between species, is unusually well conserved in Lepidoptera (Pringle *et al.* 2007; The *Heliconius* Genome Consortium 2012; Ahola *et al.* 2014; Kanost *et al.* 2016); yet there are also numerous known examples of independently evolved neo-Z and neo-W chromosomes, several of which have been well characterized cytogenetically (Traut *et al.* 2007; Yoshido *et al.* 2011; Nguyen *et al.* 2013; Šíchová *et al.* 2013; Smith *et al.* 2016). Furthermore, comparative genomic resources in this insect order are substantial and growing quickly (Challis *et al.* 2016).

In this context, we report the discovery of a neo-Z chromosome in the monarch butterfly, *Danaus plexippus*, and closely related species. Monarch butterflies, renowned for their annual migration across North America, already have a strong precedent as a model system in ecology (Urquhart 1976; Oberhauser and Solensky 2004). Recently, monarchs have emerged as a model system for genome biology, with a well-assembled reference genome, extensive population resequencing data, and a precedent for genome engineering (Zhan *et al.* 2011, 2014; Merlin *et al.* 2013; Markert *et al.* 2016). The discovery of a neo-Z chromosome further enriches the value of this species as a research model in genome biology and lays the foundation for extensive future insights into the evolution and functional diversity of sex chromosomes.

## Materials and Methods

### Sequencing coverage analysis

Illumina shotgun genomic DNA (gDNA) sequencing data for three male and three female *D. plexippus* individuals were selected for analysis from samples sequenced by Zhan *et al.* (2014). Details of sample identities, including GenBank SRA accessions, are given in Supplemental Material, Table S1 in File S1. Male–female pairs were selected on the basis of approximately equal sequencing coverage. Samples were aligned to the *D. plexippus* version 3 genome assembly with Bowtie2 (v2.1.0), using the “very sensitive local” alignment option (Langmead and Salzberg 2012; Zhan and Reppert 2013). The resulting alignments were parsed with the *genomecov* and *groupby* utilities in the Bedtools software suite (v2.17.0) to obtain a per-base median coverage depth statistic for each scaffold (Quinlan and Hall 2010). Genomic sequencing data from other *Danaus* species, also generated by Zhan *et al.* (2014), were aligned to the same assembly using Stampy (v1.0.22) (default parameters, except for *substitutionrate* = 0.1) (Lunter and Goodson 2011).

Coverage analyses comparing males and females were limited to scaffolds of lengths equal to or greater than the N90 scaffold (160,499 bp) (Zhan and Reppert 2013). Also, incomplete cases were excluded (*i.e.*, scaffolds with no reads from one or more samples). In total, 140 scaffolds were excluded, leaving 5257 scaffolds analyzed. For each sample, each scaffold’s median coverage was divided by the mean across all scaffold median coverages, thereby normalizing for differences in overall sequencing depth between samples. Samples were grouped by sex and the per-scaffold mean of normalized coverage depth was compared between sexes, formulated as the log_2_ of the male:female (M:F) coverage ratio. Autosomal scaffolds are expected to exhibit equal coverage between sexes, yielding a log_2_ ratio of zero. Z-linked scaffolds should have a ratio of one, due to the twofold-greater representation in males. Manipulation, analysis, and visualization of coverage data were performed in R (R Developement Core Team 2015).

A few scaffolds yielded intermediate M:F coverage ratios falling between equality and a twofold difference. We further examined these scaffolds using Bedtools *genomecov* to calculate per-base coverage, in order to identify regions with conspicuous shifts in coverage that may indicate potential assembly errors producing Z-autosomal chimeric scaffolds. For each sample, coverage per base was divided by the mean of all scaffold median coverage values, thus normalizing for overall sequencing depth. The normalized coverage per base was averaged within sex and visualized along the length of the scaffold by using the median of a 5-kbp sliding window, shifted by 1-kbp steps.

Point estimates for Z-autosomal break points in chimeric scaffolds were generated using a sliding window analysis of M:F coverage ratios. Putative break points were obtained as the maximum of the absolute difference between adjacent nonoverlapping windows. A window of 150 kbp with 10-kbp steps was used for DPSCF300001 and the 5′ break point of DPSCF30028. A window of 10 kbp with 1-kbp steps was used for DPSCF30044, and in a second, localized analysis between 1.5 Mb and the 3′ terminus of DPSCF30028 to localize the second, 3′ break point.

### Potential W-linked scaffolds

After the identification of the neo-Z chromosome (see *Results*), a *post hoc* search was conducted for W-linked scaffolds in the genome assembly. The *D. plexippus* genome assembly was primarily generated with data from a female individual (Zhan *et al.* 2011), thus it is possible that W-linked scaffolds are present in the genome assembly. Such scaffolds should have strongly female-biased patterns of sequencing coverage, but may also be quite short and even completely lack male coverage. We therefore reexamined the normalized coverage data as above for all scaffolds, ignoring previous criteria for length or coverage values. Scaffolds with more than twofold-greater average coverage in females were considered potentially W-linked.

Potential W scaffolds were aligned to Z-linked scaffolds via six-frame amino acid translations using the PROmer algorithm and visualized with mummerplot, both from the MUMmer software package (v3.1) (Kurtz *et al.* 2004). Predicted proteins from potentially W-linked scaffolds were searched against the all *D. plexippus* genomic scaffolds via tBLASTn and also against the GenBank nonredundant protein database via BLASTp.

### Orthology-based chromosomal assignments for D. plexippus scaffolds

Putative chromosomal linkage was predicted for *D. plexippus* scaffolds relative to the genome assemblies of three reference species, *Melitaea cinxia*, *Bombyx mori*, and *Heliconius melpomene* (the Glanville fritillary, domestic silkmoth, and postman butterfly), based on counts of orthologous genes (The International Silkworm Genome Consortium 2008; The *Heliconius* Genome Consortium 2012; Ahola *et al.* 2014). Orthologous proteins were predicted between *D. plexippus* and each reference species using the *Proteinortho* pipeline (Lechner *et al.* 2011). Using only one-to-one orthologs, we tabulated per *D. plexippus* scaffold the number of genes mapped to each chromosome in the reference species. Each *D. plexippus* scaffold was assigned to the chromosome with the highest count of orthologs in the reference species. Scaffolds were excluded from analysis when maximum ortholog count was tied between two or more scaffolds, though this situation was rare and only involved scaffolds with low genes counts.

### Point estimate of the Z-autosome fusion

The fusion point in monarch between ancestrally Z and autosomal segments was localized by aligning the homologous *H. melpomene* or *M. cinxia* chromosomes against monarch scaffold DPSCF300001 (Ahola *et al.* 2014; Davey *et al.* 2016). Alignments were based on six-frame amino acid translations using the PROmer algorithm and visualized with mummerplot, both from the MUMmer software package (v3.1) (Kurtz *et al.* 2004). We initially aligned the complete set of scaffolds from the Z (HmChr21, McChr1) or relevant autosome (HmChr2, McChr21), yielding a preliminary indication that the Z-autosome fusion point occurred at ∼4 Mbp on DPSCF300001. To refine and better visualize this phenomenon, pseudoassemblies were created for each chromosome using query scaffolds producing >500 bp of total aligned coverage on DPSCF300001. Selected query scaffolds were concatenated into a single FASTA entry, with ordering based on target alignment positions. For each species, the Z and autosomal pseudoassemblies were coaligned to DPSCF300001. The transition point between contiguous alignments of the two pseudoassemblies from distinct chromosomes was interpreted as the approximate location of the Z-autosome fusion in monarch.

### Cytogenetic analysis

All *D. plexippus* tissues used for cytogenetic analysis were from captive-bred butterflies reared on an artificial diet provided by Monarch Watch (MonarchWatch.org). Spread chromosome preparations were made from gonads of third to fifth instar larvae of both sexes following Mediouni *et al.* (2004). To test for the presence of sex chromatin, preparations of polyploid somatic nuclei were made, according to Traut *et al.* (1986), from Malpighian tubules dissected from the same material.

gDNA was isolated separately from males and females by standard phenol-chloroform extraction. Briefly, larval tissues were homogenized in liquid nitrogen, transferred in lysis buffer (100 mM NaCl, 10 mM Tris-HCl, pH 8.0, 50 mM EDTA, 100 μg/ml Proteinase K, 0.5% Sarkosyl), and incubated at 37° overnight. The samples were then treated with RNase A (10 µg/ml) and purified by three phenol, one phenol-chloroform, and one chloroform extractions. Hybridization probes were prepared from the entire complement of extracted male or female gDNA, which was labeled by aminoallyl-dUTP-Cy3 or fluorescein-12-dUTP (both Jena Bioscience GmbH, Jena, Germany) using the Nick Translation Kit (Abbott Molecular Inc., Des Plaines, IL) with 7 hr incubation at 15° (Šíchová *et al.* 2015).

Genomic *in situ* hybridization (GISH) was performed as described by Fuková *et al.* (2005). Comparative genomic hybridization (CGH) was carried out according to the protocol in Šíchová *et al.* (2013) with several modifications, as follows. Prior to denaturation, RNase A-treated slides were incubated in 5× Denhardt’s solution (0.1% Ficoll, 0.1% polyvinylpyrrolidone, 0.1% bovine serum albumin) at 37° for 30 min. A 10-µl hybridization mixture consisting of labeled female and male probes (350 ng each), sonicated salmon sperm DNA (25 µg), 50% deionized formamide, and 10% dextran sulfate in 2× SSC was denatured and allowed to reanneal at 37° for 2 hr (*cf*. Kallioniemi *et al.* 1992) before it was hybridized to the denatured female preparation.

Results were documented in a Zeiss Axioplan 2 Microscope (Carl Zeiss, Jena, Germany) equipped with the appropriate fluorescence filter set. Images were captured with an Olympus CCD Monochrome Camera XM10 equipped with cellSens 1.9 digital imaging software. The images were pseudocolored and superimposed with Adobe Photoshop CS3.

### Data availability

Putative chromosomal assignments for *D. plexippus* scaffolds and genes are provided in Table S3 in File S2. Estimated coordinates for break points reported for chimeric Z-autosomal assemblies are provided in Table S4 in File S2. Other intermediate results files and code used in the described analyses are at https://github.com/WaltersLab/Danaus_NeoZ-Public.

## Results

### Identifying Z-linked scaffolds in D. plexippus

**Figure 1:**
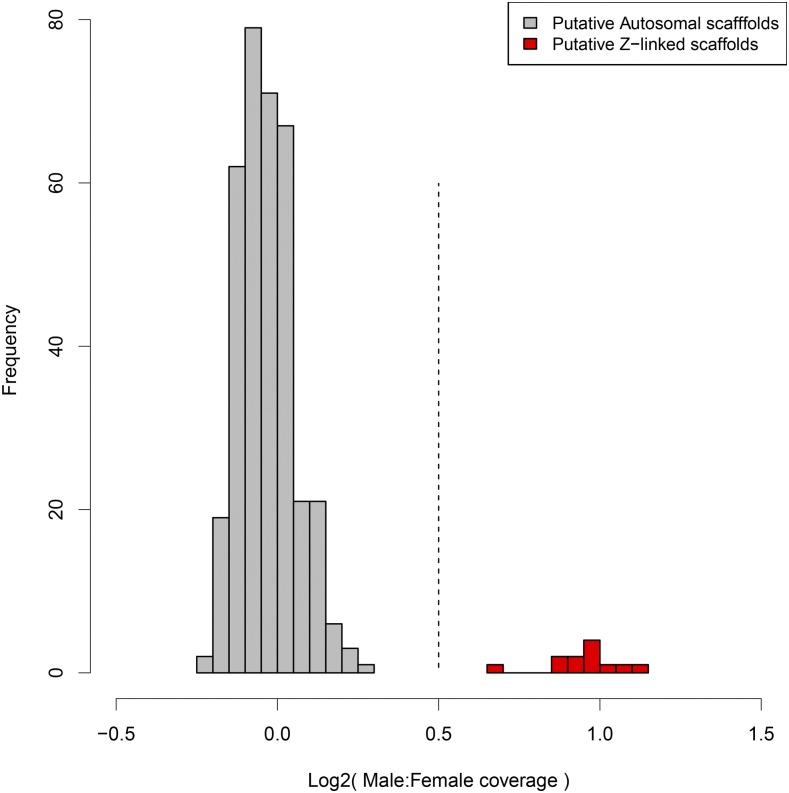
Distribution of median normalized M:F genomic sequencing coverage ratios for *D. plexippus* version 3 assembly scaffolds. Only scaffolds of length equal to or greater than the N90 scaffold are shown. The dotted line at 0.5 represents the value used to partition scaffolds as autosomal (gray) or Z linked (red).

We identified Z-linked scaffolds in the *D. plexippus* genome assembly (Zhan *et al.* 2011; Zhan and Reppert 2013) by comparing sequencing coverage from male and female samples. Males should have twice the Z-chromosome DNA content than females, while autosomes should have equal DNA content between sexes. Thus a corresponding twofold difference in sequencing coverage is expected between sexes for the Z chromosome, but not autosomes, and this can be used to identify Z-linked scaffolds (Martin *et al.* 2013; Vicoso *et al.* 2013; Mahajan and Bachtrog 2015). A histogram of M:F ratios of median coverage clearly identifies two groups of scaffolds (Figure 1). One large cluster is centered around equal coverage between sexes (log_2_ M:F = 0) and a second, smaller cluster is centered around twofold-greater coverage in males (log_2_ M:F =1). We can thus clearly distinguish the Z-linked scaffolds as those with log_2_(M:F) >0.5, with the remainder of the scaffolds presumed to be autosomal.

**Figure 2:**
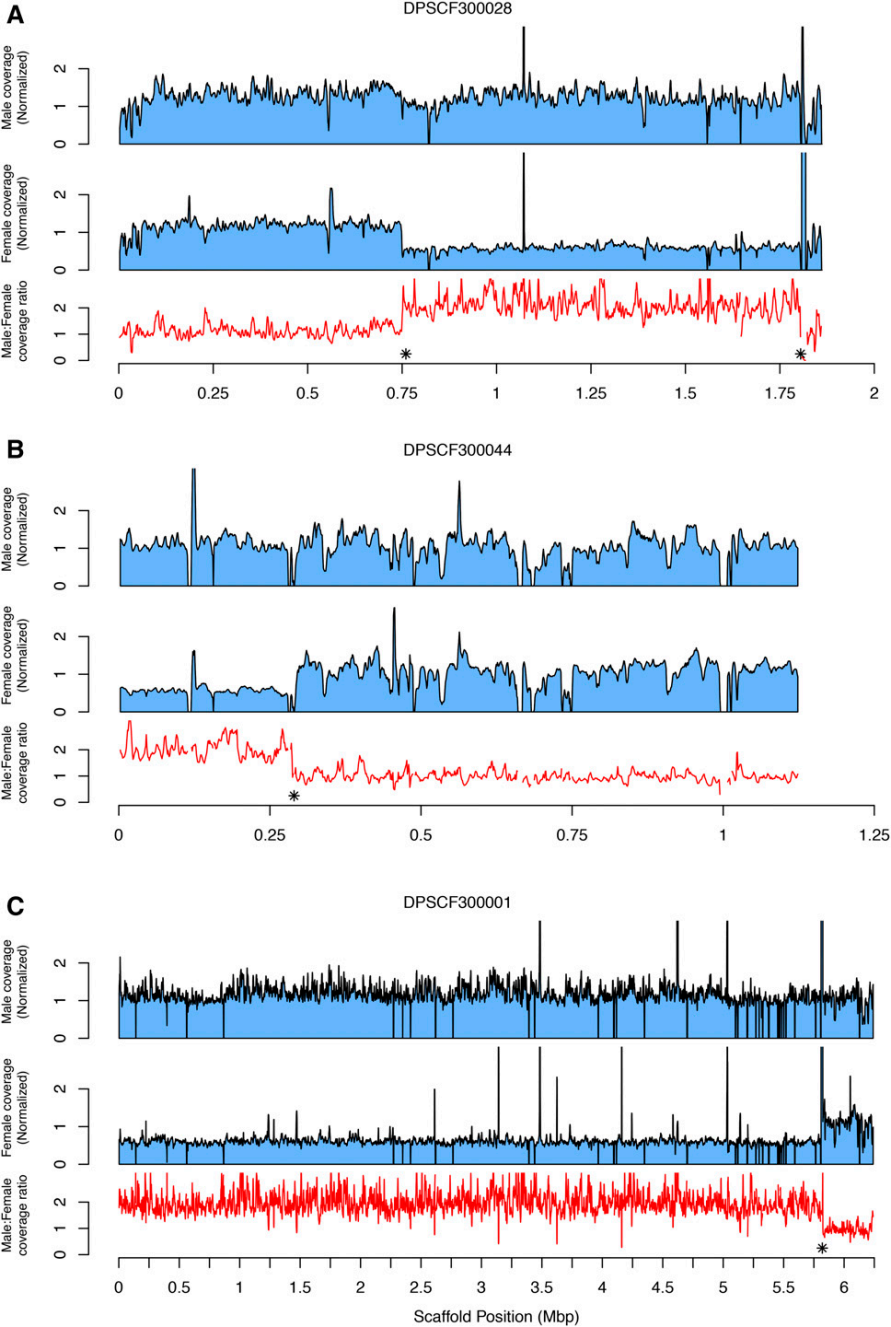
Normalized male and female coverage along the length of chimeric scaffolds, for (A) DPSCF300028, (B) DPSCF300044, and (C) DPSCF300001. Coverages are plotted as sliding windows (width = 5 kbp, step = 1 kbp) of median base pair values. The associated M:F ratio of coverage for each window is plotted as a red line below the pair of sex-specific plots. * indicates the estimated break point between Z-linked and autosomal segments of each scaffold, as determined by the maximum difference in adjacent, nonoverlapping windows of M:F ratio (see *Materials and Methods* for details).

One scaffold, DPSCF300028, appeared to have an intermediate coverage ratio, falling at log_2_ M:F ≈ 0.7. One likely explanation for such an intermediate value is that the scaffold is a chimera of Z-linked and autosomal sequence arising from an error in genome assembly (Martin *et al.* 2013). In this scenario, only a portion of the scaffold is Z-linked and gives a twofold difference in coverage between sexes; the remaining autosomal fraction of the scaffold yields equal coverage. The resulting estimate of average coverage for the entire scaffold then falls at a value between expectations for Z or autosomal scaffolds. This appears to be true for DPSCF300028, as revealed by examining base pair-level sequencing coverage across the scaffold (Figure 2A). While average male coverage is consistent across the entire length of the scaffold, female coverage exhibits a clear transition between coverage equal to males (the autosomal portion) and coverage one half that of males (the Z-linked portion). Indeed, there are two such transitions in scaffold DPSCF300028, which we estimate to occur at 0.76 and 1.805 Mbp, creating a Z segment flanked by autosomal segments.

However, an alternative explanation for this pattern could be that (portions of) the Z and W retain substantial homology and that sequencing reads from the W are mapping to closely related regions of the Z. This is particularly likely in cases where there is a recently acquired neo-sex chromosome and limited time for divergent mutations to accumulate between homologous regions of the neo-Z and neo-W. Assessing patterns of homology relative to chromosomal linkage in other species is one useful way to further assess whether these scaffolds with intermediate levels of coverage reflect chimeric assemblies or sex-linked regions of reduced divergence between (neo-) Z and W chromosomes.

### Ortholog counts link scaffolds to chromosomes

As mentioned above, Lepidoptera show a very high level of synteny conserved across substantial evolutionary distances (Pringle *et al.* 2007; The *Heliconius* Genome Consortium 2012; Ahola *et al.* 2014; Kanost *et al.* 2016). Thus, it is possible to use counts of orthologous genes to assign *D. plexippus* scaffolds to linkage groups (*i.e.*, chromosomes) delineated in other moth or butterfly species. We generated predicted orthologs between *D. plexippus* and three other reference species where genetic linkage mapping has been used to assign genomic scaffolds to chromosomes: *M. cinxia* (*n* = 31), *H. melpomene* (*n* = 21), and *B. mori* (*n* = 28) (The International Silkworm Genome Consortium 2008; The *Heliconius* Genome Consortium 2012; Ahola *et al.* 2014). *M. cinxia* and *H. melpomene* are both butterflies equally diverged from *D. plexippus* and all three are part of the same family, Nymphalidae; the silkmoth, *B. mori*, is distinctly more diverged, located outside of the suborder containing all butterflies (Wahlberg *et al.* 2009; Kawahara and Breinholt 2014).

To assign *D. plexippus* scaffolds to chromosomes, we tabulated per scaffold the counts of one-to-one reference species orthologs per reference species chromosome. *D. plexippus* scaffolds were then assigned to the reference chromosome with the maximum count of orthologs. For a few scaffolds, a tie occurred in maximum ortholog count per reference chromosome, in which case the scaffold was removed from further analysis; at most this occurred for only 14 scaffolds per reference species and usually involved small scaffolds harboring fewer than five orthologs. Typically, this method yielded a single obvious reference chromosomal assignment for each *D. plexippus* scaffold.

**Figure 3:**
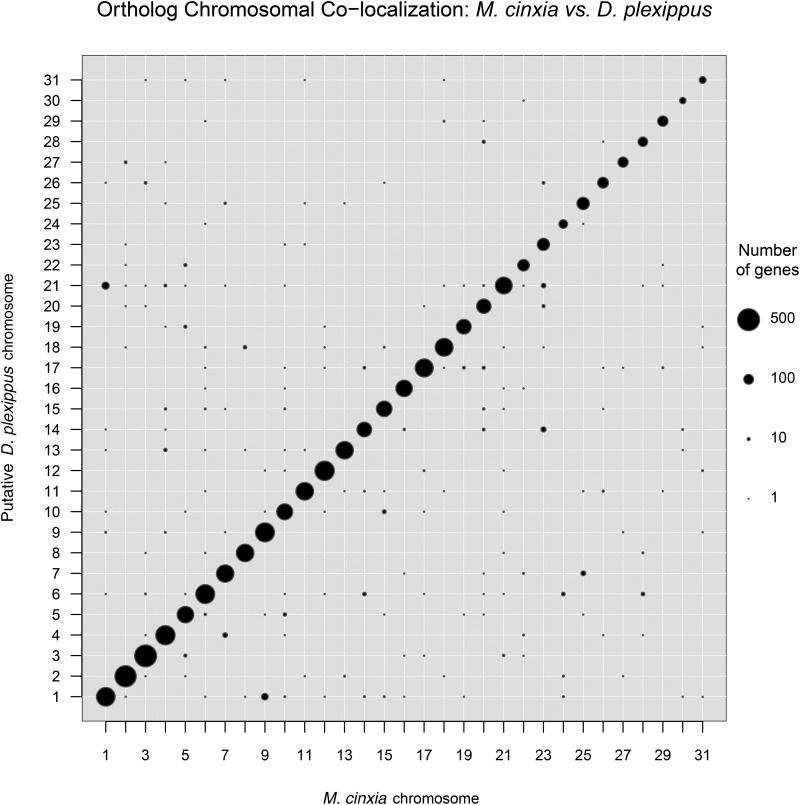
Chromosomal colinkage between *D. plexippus* and *M. cinxia* for predicted orthologous proteins.

This method of ortholog-count, chromosomal “lift-over” resulted in putative chromosomal assignments for >90% of *D. plexippus* genes relative to each reference species (Table 1 and Table S2 in File S1). Also, more than 4500 orthologous genes were colocalized to a chromosome between *D. plexippus* and each reference species. Having several thousand orthologs mapped to a chromosome in *D. plexippus* and a reference species presents the opportunity to examine the extent of chromosomal rearrangements and gene movement between the two species. Here we primarily report the comparison with *M. cinxia* because this species is believed to retain the ancestral lepidopteran karyotype of 31 chromosomes (Ahola *et al.* 2014). Furthermore, this count of chromosomes is closest to that reported for several *Danaus* butterflies, including monarch (*n* = 30, see Figure 3), making it the most appropriate comparison available (Brown *et al.* 2004). *H. melpomene* and *B. mori* are known to have less similar karyotypes involving several chromosomal fusions relative to *M. cinxia*; nonetheless, details of comparisons to these two species are reported in the supplementary content (Figure S1, Figure S2, and Table S2 in File S1) and provide comparable support for the primary findings reported here.

**Table 1:** Summary of assigning *D. plexippus* genes and scaffolds to chromosomes via orthology lift-over relative to *M. cinxia*

	No.
1:1 orthologs identified	6,740
1:1 orthologs assigned to *M. cinxia* chromosome	4,607 (68.4%)
Protein-coding genes in *D. plexippus*	15,130
*D. plexippus* protein-coding genes assigned to chromosome	14,129 (93.4%)
*D. plexippus* scaffolds putatively assigned to chromosomes	454

The cross-tabulation of chromosomal linkage for >4500 orthologs between *M. cinxia* and *D. plexippus* is summarized in Figure 3. The overwhelming majority of orthologs fall on the diagonal, suggesting substantial conservation of chromosomal linkage and relatively little gene shuffling, which appears consistent with other analyses of gene movement in Lepidoptera (The *Heliconius* Genome Consortium 2012; Ahola *et al.* 2014; Kanost *et al.* 2016). However, because this lift-over approach is based on an assumption of conserved synteny and does not include an independent assessment of chromosomal linkage in monarch, it does not exclude the possibility that large-scale changes in synteny remain to be detected. Nonetheless, two notable exceptions to the general pattern of conservation emerged, both involving the Z chromosome (Chr1). In one case (McChr9, DpChr1) we could anticipate this because of the previously identified scaffold, DPSCF300028, with intermediate-average coverage and a sharp transition in base-level depths of sequencing. This scaffold harbors 34 orthologs assigned to McChr1 and 23 orthologs assigned to McChr9. This irregular pattern of M:F coverage ratios seems most readily explained by a chimeric Z-autosome assembly, though we cannot exclude the possibility of a recent insertion from McChr9 which retains regions of substantial similarity between sex chromosomes (Figure 2A).

The second case (McChr1, DpChr21) appeared to arise entirely from a single scaffold, DPSCF300001, the largest scaffold in the *D. plexippus* v3 assembly. This scaffold carried 107 orthologs assigned to McChr21, 28 orthologs assigned to McChr1, 13 orthologs assigned to McChr23, and a few other orthologs assigned to other autosomes. Notably, despite the large number of apparently autosomal orthologs, the average M:F coverage ratio for DPSCF300001 was consistent with it being Z-linked [log_2_(M:F coverage) = 0.92]. Nonetheless, we plotted coverage across the chromosome and detected a ∼1-Mbp portion at the 3′ end of the scaffold with coverage patterns consistent with being an autosome (Figure 2C). The *M. cinxia* orthologs in this apparently autosomal portion, with an estimated break point at 5.82 Mbp, were linked exclusively to McChr23. There was not an obvious shift in sequencing coverage between sexes to indicate a misassembled Z-autosome chimera involving McChr21. Rather, it appeared that nearly the entirety of scaffold DPSCF300001 had twice the coverage in males than in females, consistent with Z linkage for regions apparently homologous both to McChr1(Z) and McChr21.

### A neo-Z chromosome in D. plexippus

**Figure 4:**
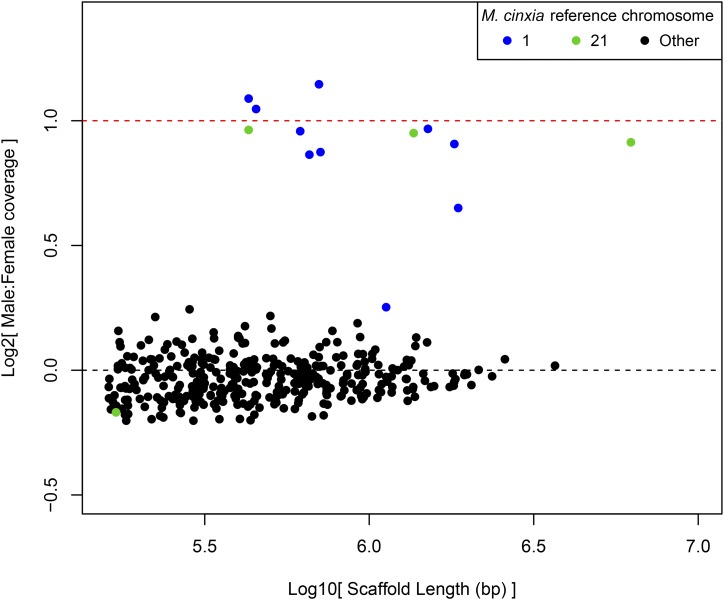
Ratios of M:F, median-normalized, genomic sequencing coverage plotted against scaffold length. Scaffolds homologous via lift-over procedure to *M. cinxia* chromosomes 1/Z (blue) and 21 (green) are plotted in distinct colors. Dotted lines indicate expected values for Z-linked (red) and autosomal (black) scaffolds.

The observation that a substantial portion of scaffold DPSCF300001 was Z-linked and homologous to McChr21, while another large section of the same scaffold was homologous to McChr1, *i.e.*, McChrZ, led us to hypothesize that a Z-autosome fusion could readily explain the karyotypic differences between *D. plexippus* (*n* = 30) and *M. cinxia* (*n* = 31). To further investigate this hypothesis of a major evolutionary transition in sex chromosome composition in the *Danaus* lineage, we examined the chromosomal assignments for all monarch scaffolds identified as Z linked via sequencing coverage ratios (Z-cov scaffolds). Specifically, we identified the unique set of reference chromosomes to which Z-cov scaffolds were assigned, and then examined the M:F coverage ratio for all scaffolds assigned to those reference chromosomes. In the case of *M. cinxia* as the reference, all Z-cov scaffolds were assigned either to McChr1 or McChr21 (Figure 4; comparable results were obtained for *H. melpomene* and *B. mori*, Figure S2 in File S1). This result provides further evidence that the Z in *D. plexippus* is a neo-sex chromosome reflecting the fusion of the ancestral Z chromosome with an autosome homologous to McChr21.

**Figure 5:**
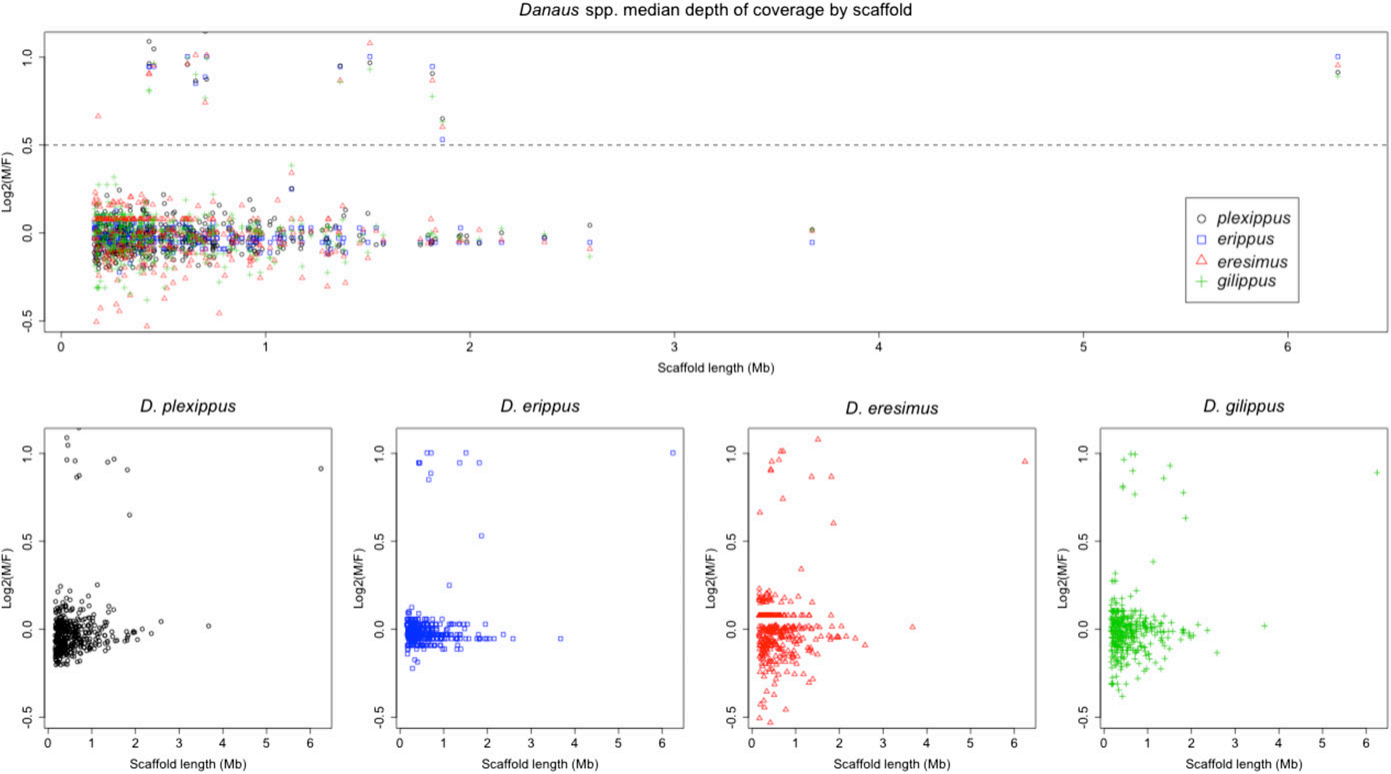
Ratios of M:F, median-normalized, genomic sequencing coverage plotted by scaffold length for four species of *Danaus* butterflies. The dotted line at log_2_(M:F) = 0.5 represents the threshold used to discern autosomal (<0.5) from Z-linked (>0.5) scaffolds.

This analysis intersecting Z-cov scaffolds with homology to *M. cinxia* revealed two scaffolds that did not fit with the expected pattern of sequencing coverage (Figure 5). First, scaffold DPSCAF300044 was assigned to McChr1(Z) but had log_2_ M:F ≈ 0.25, much more like other autosomes than other Z-linked chromosomes. This scaffold had seven Z-linked orthologs and four autosomal ones, suggesting another chimeric scaffold. Indeed, examining coverage across the scaffold revealed a clear transition in coverage as previously observed for DPSCF300001 and DPSCF300028 (Figure 2B). Thus the low M:F coverage ratio for this scaffold could be the artifact of an assembly error. Alternatively, the M:F coverage ratio could reflect reduced divergence between neo-Z and neo-W, with neo-W-linked reads mapping to the Z scaffold. Examining base-level coverage, we were again able to identify a sharp transition in coverage levels that partitioned the scaffold into two sections. One section was consistent with autosomal (*i.e.*, equal) M:F coverage and the other with Z-linked (*i.e.*, twofold) M:F coverage, with a break point estimated at 0.29 Mbp from the 5′ end. The autosomal section contained approximately equal counts of orthologs assigned to two distinct autosomes in *M. cinxia*, as well as in the other reference species analyzed, so linkage to a specific autosome could not be predicted. Nonetheless, homology to autosomes other than McChr21 for the portion of DPSCAF300044 with equal M:F coverage supports the notion of a chimeric assembly over retained allosome homology. Table S3 in File S2 summarizes break points and predicted scaffold assignments for the three chimeric Z-autosome scaffolds identified here.

DPSCF300403 was the other scaffold tentatively identified as homologous to McChr21, but without the characteristic twofold difference in M:F coverage. Rather, the M:F coverage ratio along the scaffold was approximately equal, consistent with it being entirely autosomal (Figure S3 in File S1). However, it may also be that retained sex-linked homology could produce this coverage pattern. In this case, the scaffold carried only a single one-to-one orthologous gene (and only five protein-coding genes in total), so the assignment to McChr21 is rather tenuous. This scaffold also had a single one-to-one ortholog found in *B. mori*, and none identified in *H. melpomene*. Given the quite limited evidence of chromosomal homology for this scaffold, we thus consider DPSCF300403 essentially uninformative concerning the presence of neo-sex chromosomes in *D. plexippus*.

### The neo-Z chromosome exists in the monarch’s close relatives

The monarch population genomic data set from Zhan *et al.* (2014) also contained male and female resequencing samples from three closely related congeners: *D. gilippus*, *D. erippus*, and *D. eresimus*. This allowed us to assess whether this neo-Z exists in these related species similarly to *D. plexippus*. Analyzing male *vs.* female resequencing in these species does indeed show the same scaffolds homologous to both McChr1 and McChr21 as having coverage differences consistent with a neo-Z (Figure 5). Thus it appears that the origin of this neo-Z predates the diversification of the genus *Danaus*.

### Annotating chromosomal linkage

The combination of sequencing coverage analysis and comparative lift-over allowed us to provisionally assign most genes to chromosomes in *D. plexippus*. Genes falling on Z-cov scaffolds, or within the portion assessed as Z linked for noted chimeric scaffolds, have been assigned to the Z chromosome. We further partitioned these Z-linked genes into being on the ancestral (anc-Z) or neo- (neo-Z) portion of the Z, based on scaffold homology to reference chromosomes. In the case of DPSCF300001, we localized the fusion point between anc-Z and neo-Z by aligning *M. cinxia* and *H. melpomene* scaffolds from the Z (HmChr21, McChr1) or relevant autosome (HmChr2, McChr21). Alignments with both species were consistent in placing the fusion point at ∼3.88 Mbp from the 5′ end of the scaffold (Figure S4). Otherwise, genes and scaffolds were assigned to chromosomes based directly on the results of the lift-over relative to *M. cinxia*. Table 2 gives a tabulated summary of results, while results for every protein-coding gene are provided in Table S4 in File S2.

**Table 2:** Summary of provisional chromosomal linkage for *D. plexippus* protein-coding genes, with chromosomal identity reflecting homology to *M. cinxia*

Chromosome	Number of Genes
1(Z)	1101
Anc-Z	624
Neo-Z(21)	477
2	704
3	758
4	582
5	494
6	689
7	483
8	535
9	647
10	452
11	574
12	576
13	501
14	429
15	493
16	524
17	604
18	561
19	414
20	399
22	302
23	318
24	185
25	329
26	274
27	250
28	284
29	294
30	151
31	168
Not assigned	1055

### Cytogenetic analysis

**Figure 6:**
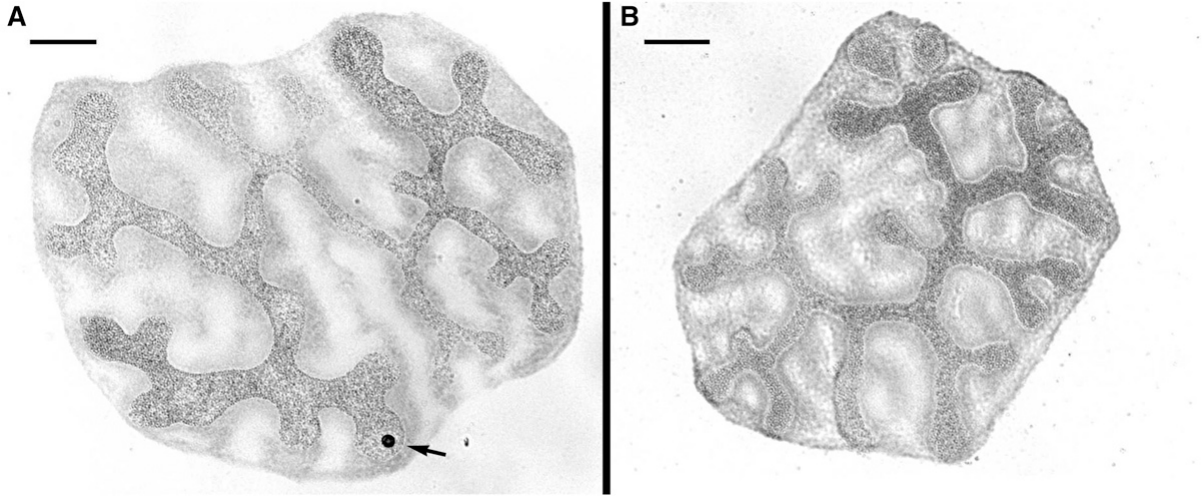
Multi-lobed, highly polyploid nuclei of the Malpighian tubules from *D. plexippus* larvae stained by orcein. (A) A female nucleus with a single deeply stained sex chromatin body (arrow). (B) A male polyploid nucleus with no heterochromatin. Bar, 20 µm.

Preparations of highly polyploid nuclei of Malpighian tubules from *D. plexippus* larvae were examined for the presence of a so-called “sex chromatin,” *i.e.*, a female-specific heterochromatin body consisting of multiple copies of the W chromosome (Traut and Marec 1996). Large multi-lobed nuclei were observed on both male and female preparations (Figure 6), which suggests a high degree of polyploidy in the examined cells, as expected in Malpighian tubules (*cf*. Buntrock *et al.* 2012). All female nuclei contained a single, highly stained heterochromatin body (Figure 6A). In contrast, no such heterochromatin was detected in male somatic nuclei (Figure 6B). This discrepancy confirms the female specificity of the heterochromatin observed, and indicates a W chromosome is a component of the *D. plexippus* genome.

**Figure 7:**
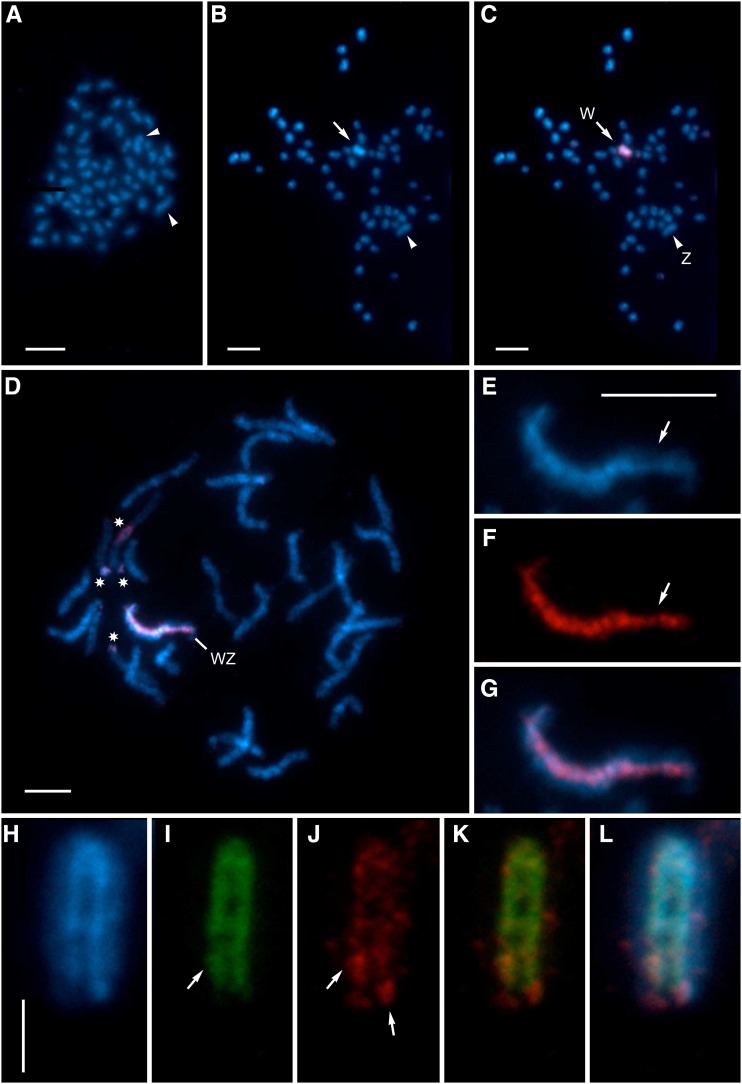
Cytogenetic analysis of sex chromosomes in *D. plexippus*. Chromosomes were counterstained by DAPI (blue). (A) Male mitotic metaphase complement consisting of 2*n* = 60 elements. Note the large chromosome pair (arrowheads). (B) Female mitotic metaphase nucleus comprising 2*n* = 60 chromosomes. The largest elements differ in intensity of their staining (arrowhead and arrow, the latter marking highly stained chromosome). (C–G) Female complements examined by GISH. Female-derived probe was labeled by Cy3 (red). (C) The same mitotic complement as in (B). The probe identified the DAPI-positive chromosome as a W chromosome and thus indicated the other large chromosome to be the Z chromosome. (D) Female pachytene complement consisting of 30 bivalents. The WZ bivalent was clearly recognized by female-derived probe, which highlighted whole W chromosome except its terminal segments. The probe also marked three terminal regions and one interstitial region of several autosomes (*). (E) The WZ bivalent stained by DAPI. Note that only about two thirds of the W chromosome thread are deeply stained and apparently heterochromatic. The more lightly stained euchromatic segment is marked by an arrow. (F) Hybridization signal of female-derived probe highlighting the W chromosome thread. Note that the signal is weaker in the euchromatic region (arrow). (G) Composite image of DAPI and the probe. (H–L) A pachytene WZ bivalent probed by CGH. Male-derived probe was labeled by Cy3 (red), female-derived probe by fluorescein (green). (H) The WZ bivalent stained by DAPI. The weakly stained Z is wrapped around the strongly stained W, which is folded in half. (I) Hybridization signal of female-derived probe. Note that the probe labeled only a small region of the euchromatic segment (arrow). (J) Hybridization signal of male-derived probe strongly highlights two W segments (arrows). (K) Both the female- and male-derived signals merged. (L) Composite image of DAPI and both probes. Bars, 5 µm in (A–G) and 2.5 µm in (H); (E–G) and (H–L) have the same scale.

Spread mitotic complements of males contained 2*n* = 60 chromosomes. The chromosomes were generally small and uniform, as is typical of lepidopteran karyotypes (Marec *et al.* 2009), except for two distinctly larger elements (Figure 7A). Female mitotic metaphase consisted of 2*n* = 60 elements as well. In females, however, the two largest chromosomes differ in intensity of their DAPI staining. The deeply stained element found exclusively in females presumably represents the W sex chromosome consisting of AT-rich heterochromatin (Figure 7B; Kapuscinski 1979).

To identify the W chromosome, we used GISH in which a probe generated by fluorescently labeling female gDNA is hybridized to female chromosome preparations in presence of excess of unlabeled male competitor gDNA. The male competitor gDNA binds probe sequences common to both sexes (*e.g.*, autosomal and Z linked) and does not interact with the prepared sample. The remaining female gDNA probe hybridizes to regions containing female-specific and -enriched sequences, clearly differentiating the W chromosome in both mitotic and pachytene nuclei (Figure 7, C and D). GISH confirmed that the W chromosome is one of the two exceptionally large chromosomes in the *D. plexippus* karyotype (Figure 7C). In pachytene oocytes, the WZ bivalent was easily discernible by the heterochromatic W chromosome thread. However, about one third of the chromosome was not strongly highlighted by DAPI and the intensity of its staining was comparable to autosomes, indicating a substantially euchromatic portion of the W that is consistent with being a neo-W (Figure 7E). Accordingly, the female-derived probe did not highlight the W chromosome homogeneously; the signal was weaker on both its ends and the euchromatic segment (Figure 7, E and F). Female-derived probes in GISH also strongly labeled one interstitial and a few terminal regions of some autosomes, which most likely contain clusters of repetitive sequences (Figure 7D).

CGH is used to assess the broad molecular composition of the W chromosome (Sahara *et al.* 2003). It is based on the same principle as GISH. In this method, however, the same amount of male and female gDNA labeled by Cy3 and fluorescein, respectively, is hybridized to preparations. In *D. plexippus*, the hybridization signal of female-derived probe labeled by fluorescein was largely consistent with the results obtained by GISH. The signal highlighted nearly the entire W chromosome thread, with the exception of its termini and euchromatic segment, in which the probe detected only a small interstitial block (Figure 7, I, K, and L). The male-derived probe labeled by Cy3 provided a relatively weak hybridization signal, which was scattered along the W chromosome. This male probe highlighted only two regions: the W chromosome end opposite to the euchromatic segment and the region highlighted within the euchromatin by the female-derived probe (Figure 7, J–L). Both probes also detected the same autosomal regions as GISH (data not shown).

### Potential W scaffolds

In searching for W-linked scaffolds, we were primarily concerned with identifying sequences that would be informative regarding the presence of a neo-W chromosome. We identified 12 scaffolds with average coverage patterns suggestive of W-linkage. These scaffolds ranged in length from ∼4 to 180 kbp and collectively harbored a total of 17 predicted protein-coding loci. However, none of the scaffolds showed obvious homology to any Z-linked scaffolds. Only six of the 17 loci produced significant (e-value <10^−5^) tBLASTn hits to any Z-linked scaffold. In each case, the protein also had at least one, and typically many, stronger hits to autosomal loci. Thus, of primary significance concerning the presence of a neo-W chromosome, none of these potentially W-linked scaffolds showed evidence of homology to Z-linked scaffolds. A comprehensive description of results is provided in File S3.

## Discussion

Using a combination of genomic resequencing, comparative genomics, and cytogenetic analysis, we have documented the presence of a neo-Z chromosome in *Danaus* butterflies, along with what is likely an accompanying neo-W chromosome. This discovery of neo-sex chromosomes in *Danaus* butterflies and our discrimination of genes falling on the ancestral *vs.* recently autosomal portions of the Z are fundamental observations that provide the foundation for a host of future inferences. These results create novel opportunities to address rates of molecular evolution, the evolution of dosage compensation, the pattern of allosome divergence, and many other important questions in sex chromosome biology, all in a female-heterogametic species that is also an emerging genomic model system.

In analyzing patterns of chromosomal fusion in *H. melpomene* and *B. mori* relative to *M. cinxia*, Ahola *et al.* (2014) report a significant tendency for a limited set of ancestral chromosomes, particularly the smallest ones, to be involved in chromosomal fusion events. Neither the ancestral Z nor McChr21 are among these small, repeatedly fused chromosomes; thus the chromosomal fusion reported here does not fit neatly with this pattern. Nonetheless, HmChr2 (homologous to McChr21) is the second-smallest chromosome that remains unfused between these lineages (Davey *et al.* 2016). Consequently, it is also difficult to argue strongly that this Z-autosome fusion in *Danaus* is a striking contrast to the trend of chromosomal fusions involving small chromosomes.

Motivated by the bioinformatic discovery of a neo-Z chromosome, we performed cytogenetic analysis of the *D. plexippus* karyotype to provide further insight into evolution and molecular composition of the monarch sex chromosomes. Previously, an observation of *n* = 30 chromosomes was reported only for males (Nageswara-Rao and Murty 1975). [See Price (2017) for potential controversy over *D. plexippus* chromosome number, but note that Brown *et al.* (2004) states that “the number for *D. plexippus* comes from a population kept at the University of Madras, India.” This note was overlooked by all and offers plausible explanation for *D. plexippus* in India.] Our current analysis confirms the same chromosome number not only in males but also in females (Figure 7, A and B). Furthermore, detailed analysis of mitotic complements revealed a large chromosome pair (Figure 7, A and B) and GISH clearly identified one chromosome of the pair as the W chromosome (Figure 7C). A similar, extraordinarily large chromosome pair was recently shown to correspond to neo-sex chromosomes in leaf-roller moths of the family Tortricidae (Nguyen *et al.* 2013; Šíchová *et al.* 2013).

GISH represents a simplified version of CGH, which has been successfully used for evaluating the gross molecular composition of lepidopteran W chromosomes (*e.g.*, Mediouni *et al.* 2004; Fuková *et al.* 2005). Previous studies in several moth species contrasted fluorescence intensities of male- *vs.* female-derived probes and identified two common types of repeats on lepidopteran W chromosomes: (i) repetitive sequences common to both males and females, *i.e.*, present in autosomes and Z chromosome; and (ii) repetitive sequences exclusively or predominantly present in females (Sahara *et al.* 2003). In these previously studied species, the W primarily contains the first type, *i.e.*, ubiquitous repeats (*e.g.*, Fuková *et al.* 2005; Šíchová *et al.* 2013). In contrast, the monarch W appears distinct from the W chromosomes of these other species because the majority of the monarch W chromosome overwhelmingly contains repeats of the second type, *i.e.*, female-limited repeats. The monarch W was labeled primarily by the female-derived probe (Figure 7, I–K), indicating that it is primarily comprised of repetitive sequences either specific to or greatly enriched on the W chromosome. Only two small segments of the W showed notably high densities of ubiquitous repeats commonly enriched on the entire W in other lepidopteran species.

This discrepancy between monarch and other species could be related to the relatively small size of the *D. plexippus* genome. The monarch butterfly represents the smallest lepidopteran genome yet sequenced, with haploid nuclear content of male being 284 Mbp (see Doležel *et al.* 2003 for the conversion of pg of DNA to Mbp; Gregory and Hebert 2003) and female being 273 Mbp (Zhan *et al.* 2011). This small genome is presumably depleted of repetitive sequences found more ubiquitously among the autosomes and Z in other Lepidoptera. Indeed, repeat content constitutes only 13.1% of the *D. plexippus* genome assembly (Zhan *et al.* 2011). The results of CGH thus could reflect the fact that the W chromosome represents the last refuge for many such repetitive sequences in the monarch genome after otherwise being purged from the genome.

Cytogenetic analyses further indicate that the monarch W chromosome exhibits notable compositional heterogeneity. Both GISH and CGH revealed terminal gaps in female-derived signal (Figure 7, G and L). One plausible explanation for this lack of female-specific signals on the monarch W chromosome ends is that ectopic recombination homogenizes these subtelomeric chromosome regions. A similar GISH result was obtained in the codling moth, *Cydia pomonella* (Tortricidae), where the female-derived probe labeled the entire W chromosome except for both subtelomeric segments (Fuková *et al.* 2005). A related observation was made by Van’t Hof *et al.* (2013), who proposed that a copy of the Z-linked *laminin A* gene was transferred to and maintained on a W chromosome of the peppered moth, *Biston betularia* (Geometridae), by gene conversion resulting from ectopic recombination between repeats localized in terminal chromosome regions. Thus it seems that this pattern of reduced differentiation in subtelomeric regions of the W chromosome is a relatively common phenomenon in Lepidoptera.

Another region distinctly identified by CGH corresponds to an interstitial block localized within a euchromatic chromosome segment. The block was illuminated by both female- and male-derived probes (Figure 7,J–L), which suggests presence of repetitive sequences common to autosomes and the Z chromosome (Sahara *et al.* 2003). This block, together with the adjacent terminal region, forms a chromosome segment with distinct molecular composition comprising about one third of the W chromosome. The monarch W thus shows a bipartite organization, with only two thirds of the chromosome being highly heterochromatic, while the remaining third appears euchromatic.

The identification of the neo-Z and putative neo-W in *Danaus* butterflies raises several questions about how, and when, these sex chromosomes arose. Concerning the sequence of events giving rise to the current karyotype, we can identify three possible scenarios resulting in equal chromosome numbers in males and females of *D. plexippus*. The first two scenarios begin with the fusion of the Z chromosome to the autosome corresponding to McChr21, giving rise to a multiple sex chromosome system of W_1_W_2_Z, and then followed either by (i) the loss of one W chromosome, or (ii) by the fusion of the W chromosomes. Alternatively, (iii) fusion of the ancestral W chromosome with the autosome corresponding to McChr21 resulted in a sex chromosome constitution of WZ_1_Z_2_ and was followed by fusion of the Z chromosomes. Given the large size of the monarch W chromosome relative to chromosomes other than the Z, which is of comparably large size, the first scenario would further demand considerable expansion of the W chromosome sequence. The observed combination of size and bipartite organization of the *D. plexippus* W chromosome seems more parsimonious with scenarios (ii) or (iii), *i.e.*, a neo-W chromosome resulting from a W-autosome fusion occurring in addition to the neo-Z formation. However, caution is advised in this interpretation because W chromosome size can be misleading (Schartl *et al.* 2016) and cytogenetic examination of species bearing neo-Z chromosomes revealed considerable differences in the structure of their W chromosomes (Šíchová *et al.* 2013).

In the hope of further supporting this neo-W hypothesis, we investigated the possibility that W-linked scaffolds were present in the *D. plexippus* genome assembly [which was generated from a female sample (Zhan *et al.* 2011)]. Crucially, identifying W-linked sequence with obvious homology to the neo-Z would strongly indicate the presence of a neo-W chromosome. Unfortunately, we found no evidence for any such scaffolds; none of the potentially W-linked scaffolds identified via coverage patterns exhibited any notable similarity to Z-linked scaffolds. It is also possible that sufficient similarity exists between the neo-Z and a neo-W that substantial portions of the two would be collapsed into a single scaffold during genome assembly. For these regions/scaffolds, the M:F sequencing coverage would be strongly shifted toward equality. We did identify a few scaffolds with some homology to the Z or the fused autosome (*e.g.*, McChr21) that presented intermediate-average coverage levels, with particular regions of nearly equal coverage between sexes. However, in almost every case, the region with balanced coverage also exhibited homology to chromosomes other than the one involved in the Z-fusion event. This is seemingly better explained as chimeric scaffolds resulting from assembly errors than by extensive allosome homology. The only potential exception to this was the ambiguous case of DPSCF300403, which could not be robustly assigned to a homologous chromosome. Ultimately, we could not identify any portion of the *D. plexippus* genome assembly that strongly supported the presence of a neo-W chromosome.

Nonetheless, these negative results are far from sufficient to exclude the presence of a neo-W in *Danaus*. If the neo-W is substantially degenerate and repeat rich, as seems to be the case, it would have been quite difficult to assemble, and thus very poorly represented, given the methods used for the current *D. plexippus* genome assembly. Nonetheless, determining whether and what protein-coding genes may remain on the W is of particular interest in this system. A promising route to further evaluate the presence of a neo-W would be via the application of novel long-read sequencing technologies and targeted laser capture of the W chromosome (Fuková *et al.* 2007; Chang and Larracuente 2017; Tomaszkiewicz *et al.* 2017).

Concerning the timing of these fusion events, our results suggest that the neo-sex chromosome system was present in a common ancestor of the four species examined, namely *D. plexippus*, *D. gilippus*, *D. erippus*, and *D. eresimus*. Given the phylogenetic relationships between *Danaus* species (Brower *et al.* 2010) and their chromosome numbers (Brown *et al.* 2004), the sex chromosome–autosome fusion appears to be a shared trait for the genus *Danaus* and therefore occurred at least 5 MYA (Lushai *et al.* 2003). However, we cannot exclude the possibility that the neo-sex chromosomes evolved even earlier in the evolution of the subtribe Danaini, as the karyotype *n* = 30 is often found elsewhere in the subtribe, shared by several species of the genera *Danaus*, *Euploea*, *Idea*, and *Lycorea* (Maeki and Ae 1968; Brown *et al.* 2004; Ahola *et al.* 2014). Nonetheless, assuming a minimum age of 5 MYA is helpful for contextualizing the *Danaus* neo-sex chromosomes in light of molecular analyses from other derived sex chromosome systems that are better analyzed.

Arguably the best comparison to neo-sex chromosomes in Lepidoptera is found among *Drosophila* fruit flies, which provide multiple cases of thoroughly studied neo-sex chromosomes of various ages. Importantly, in both *Drosophila* and Lepidoptera, meiosis in the heterogametic sex is achiasmatic, *i.e.*, it occurs without recombination. Recombination between neo-sex chromosomes is thus restricted immediately and completely upon sex chromosome–autosome fusion and there is no opportunity for the formation of evolutionary strata or pseudoautosomal regions. For comparison, an age of 5 MY would place the *Danaus* neo-W roughly midway between the ages of the neo-Y chromosomes found in *Drosophila pseudoobscura* and *D. miranda*. We might therefore expect at least somewhat less degeneration on the *Danaus* neo-W than the *D. pseudoobscura* neo-Y, which formed in the range of 10–18 MYA and is now entirely heterochromatic with almost no detectable homology to the neo-X and only a handful of functional genes remaining (Carvalho and Clark 2005; Bachtrog 2013). In contrast, after (at least) 5 MY, degeneration of the *Danaus* neo-W chromosome has likely progressed far beyond the stage exemplified by the neo-Y of *D. miranda*, which formed ∼1 MYA. The *D. miranda* neo-Y chromosome is partially heterochromatic and has massively accumulated repetitive sequences. While the majority of neo-X-linked genes still have homologs on the neo-Y chromosome, nearly half of them are already pseudogenes (Zhou and Bachtrog 2012; Bachtrog 2013). Notably, Zhou and Bachtrog (2012) reported M:F ratios of read mapping coverage along the neo-X chromosome to be ∼3:4. In *D. plexippus*, sequencing data yield a very consistent 2:1 M:F coverage ratio on scaffold regions corresponding to McChr21. If a neo-W retained substantially close homology to the neo-Z, we would expect many sequencing reads emanating from the neo-W to align to the neo-Z, and shift this coverage ratio toward one. This evidently does not occur, suggesting substantial divergence between the neo-Z and any homologous neo-W sequence that is retained. The apparent absence of any neo-W scaffolds in the current genome assembly is consistent with substantial degeneration of a neo-W, assuming one exists.

Cytogenetic analyses confirm that the *D. plexippus* W chromosome is well differentiated relative to the Z (Figure 7, D–G). However, the presence of euchromatin on the W chromosome is intriguing and it is tempting to interpret its presence as indirect evidence of transcriptional activity of W-linked McChr21 orthologs. In general, heterochromatinization of a neo-W/Y chromosome involves two processes: (i) spreading of repetitive sequences which could serve as nucleation sites for initiating heterochromatin formation (Stanojcic *et al.* 2011); and (ii) loss of expression of W-linked genes, since expression generally attenuates the spread of heterochromatin (*cf*. Wheeler *et al.* 2009). Thus, presence of euchromatin could be due to low accumulation of certain types of repeats rather than ongoing gene expression. Analyses of contiguous W chromosome sequences are necessary to determine whether and how the protein-coding sequences are preserved on the W chromosome of *D. plexippus* (*cf*. Carvalho *et al.* 2015).

Finally, it should be noted that a relatively modern W-autosome fusion was recently reported to be segregating in the African queen butterfly, *D. chrysippus*, where it controls color pattern and male killing and is driving population divergence across a hybrid zone (Smith *et al.* 2016). Thus, the W-autosome fusion in *D. chrysippus* may represent a compound neo-W involving two distinct former autosomes. This pattern of relatively frequent karyotypic changes within the genus further recommends *Danaus* butterflies as an excellent model system for studying sex chromosome evolution.

### Conclusion

We have used a combination of genome sequencing coverage, comparative genomic analysis, and cytogenetics to demonstrate that *Danaus* butterflies harbor a neo-Z chromosome resulting from the fusion of the ancestral Z chromosome and an autosome homologous to Chr21 in *M. cinxia*. Also, at least in the case of monarch butterflies, it appears plausible that a separate fusion event involving the W and this same autosome has resulted in a large neo-W chromosome with a prominent euchromatic region. Our analysis also identified and resolved several Z-autosome chimeric scaffolds in the most recent assembly of the *D. plexippus* genome. This discovery and provisional assignment of chromosomal linkage for >90% of *D. plexippus* genes paves the way for myriad and diverse investigations into sex chromosome evolution, which are likely to be of distinct importance given the increasing prominence of *Danaus* butterflies as a female-heterogametic model species for functional and evolutionary genomics.

## Supplementary Material

Supplemental material is available online at www.g3journal.org/lookup/suppl/doi:10.1534/g3.117.300187/-/DC1.
