# Supplementary material for: Neo-sex Chromosomes in the Monarch Butterfly, Danaus plexippus
Source: G3 (Bethesda). 2017 Aug 23;7(10):3281–94. doi: 10.1534/g3.117.300187 (PMC5633379; doi:10.1534/g3.117.300187)
Supplement: Supplementary file 1 [file 3281FigureS4.pdf]

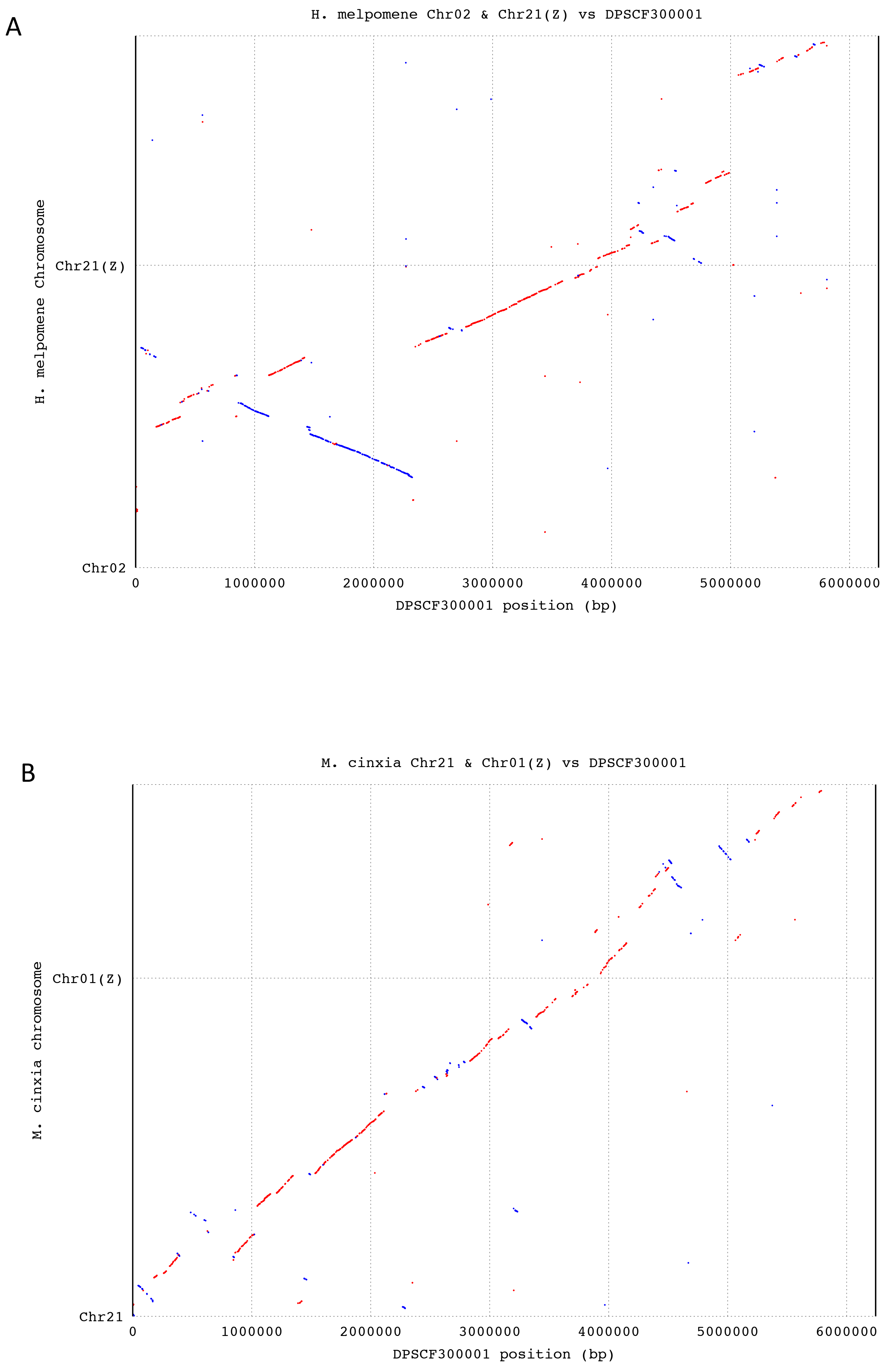

Figure S4. Promer alignments of DPSCF300001 against the Z and homologous autosome from (A) *H. melpomene* and (B) *M. cinxia*. Best one-to-one alignments were generated using default parameters. Manual inspection of alignment coordinates revealed the transition on DPSCF300001 from neo-Z to anc-Z occurs in a window between positions 3.878 and 3.886 Mbp.
