# Supplementary material for: Neo-sex Chromosomes in the Monarch Butterfly, Danaus plexippus
Source: G3 (Bethesda). 2017 Aug 23;7(10):3281–94. doi: 10.1534/g3.117.300187 (PMC5633379; doi:10.1534/g3.117.300187)
Supplement: Supplementary file 3 [file 3281FileS1.pdf]

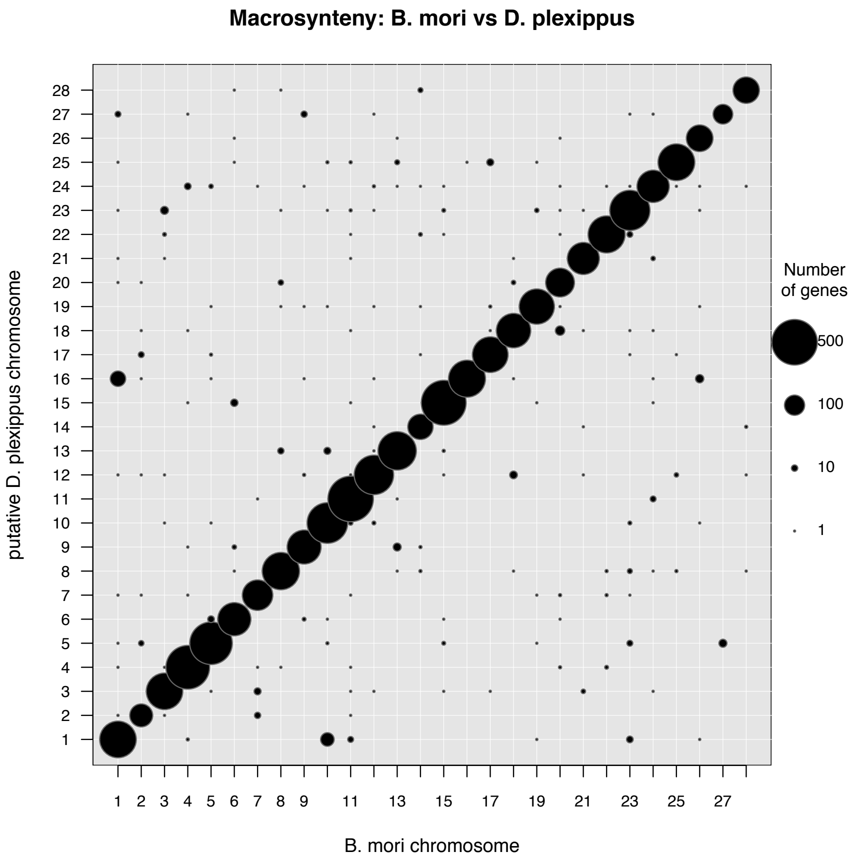

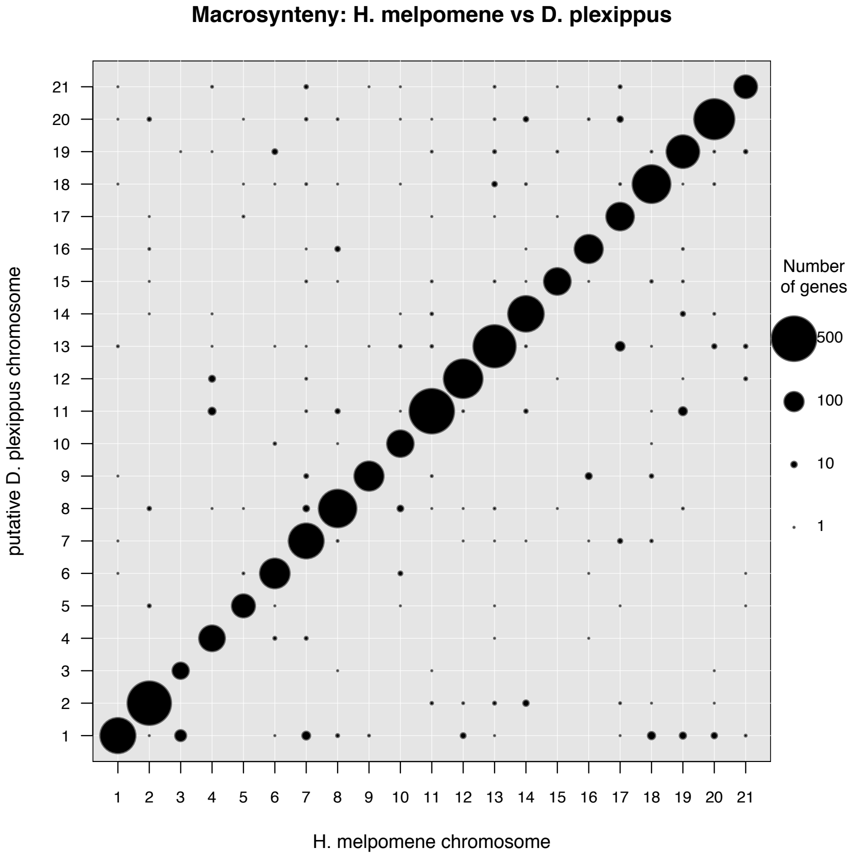

Figure S1. Chromosomal co-linkage between *D. plexippus* and *B. mori* (top) or *H. melpomene* (bottom) for predicted orthologous proteins.

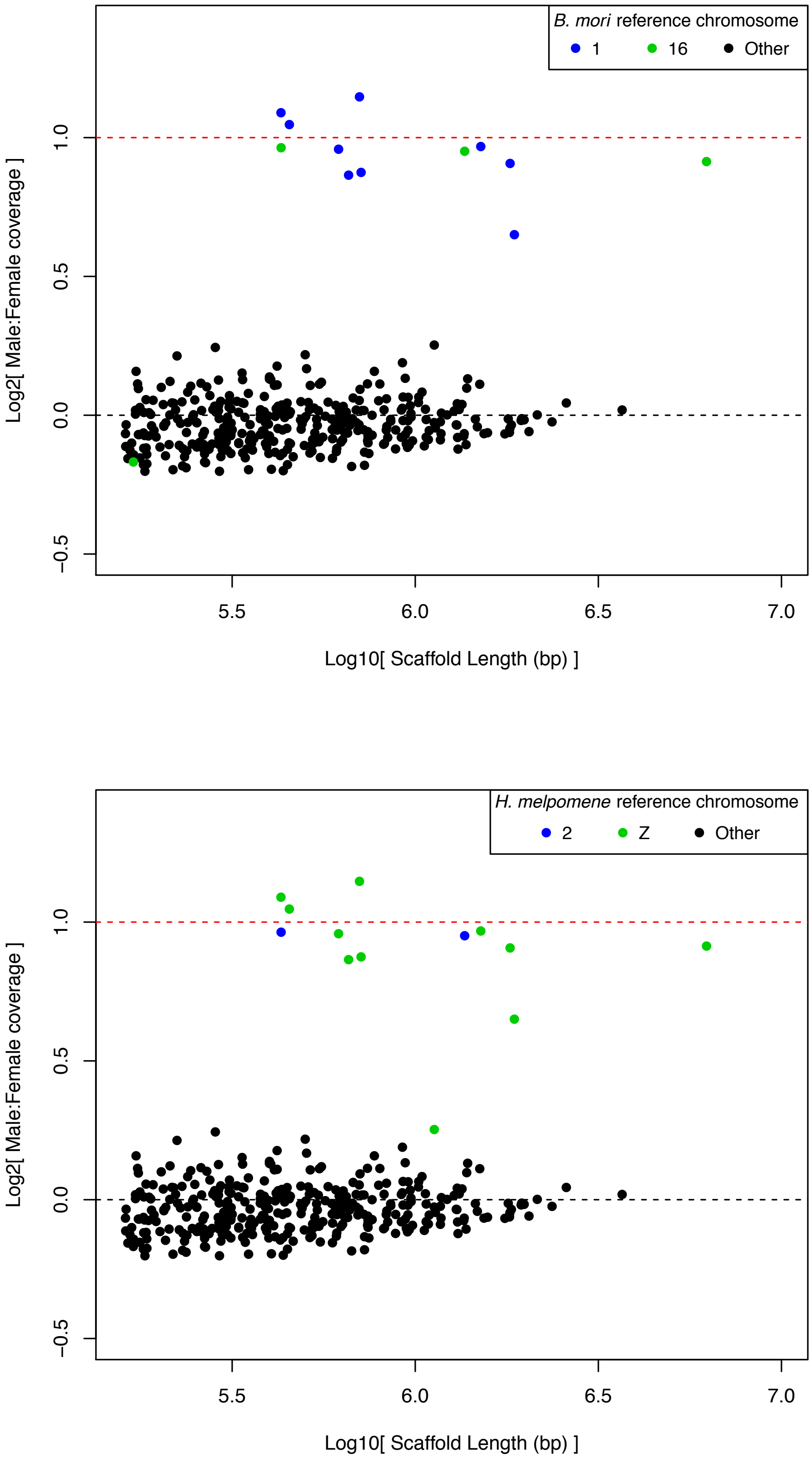

Figure S2. Ratios of male:female median normalized genomic sequencing coverage plotted by scaffold length. Scaffolds assigned to chromosomes putative homologous to the neo-Z chromosome in *D. plexippus* are plotted in distinct colors. Top, relative to *B. mori*, to chromosomes 1 (*i.e.,* Z ; blue) and 16 (green). Bottom, relative to *H. melpomene*, chromosomes 1 (*i.e.,* Z ; green) and 2 (blue).

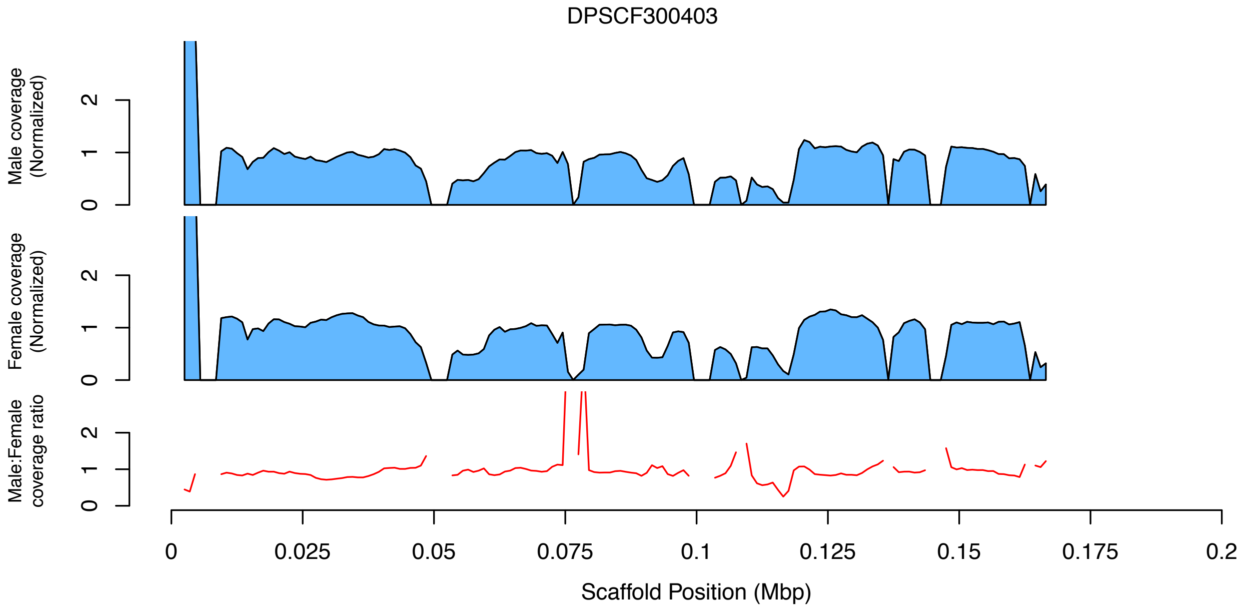

Figure S3. Normalized male and female coverage along the length DPSCF300403. Coverages are plotted as sliding windows (width = 5Kbp, step = 1 Kbp) of median basepair values. The associated male:female ratio of coverage for each window is plotted as a red line below the pair of sex-specific plots.

Supplementary Table S1. Sample identification details for sequencing data used in coverage analyses.

Region	Sample	Species	Sex	Accession
North America	Plex_MA_HI004_M	*plexippus*	male	SRX679269
	Plex_MA_HI035_F	*plexippus*	female	SRX679310
	Plex_FLn_StM123_F	*plexippus*	female	SRX680105
	Plex_FLn_StM146_M	*plexippus*	male	SRX681753
	Plex_WSM_M36_M	*plexippus*	male	SRX680118
	Plex_WSM_M38_F	*plexippus*	female	SRX681528
Other *Danaus*	Erip_BRA_16005_F	*erippus*	female	SRX682069
	Erip_BRA_16008_M	*erippus*	male	SRX682070
	Eres_CRC_92_F	*eresimus*	female	SRX682071
	Eres_FL_27_M	*eresimus*	male	SRX682072
	Gili_CRC_30_M	*gilippus*	male	SRX682073
	Gili_TX_01_F	*gilippus*	female	SRX998564

Table S2. Summary of assigning *D. plexippus* genes and scaffolds to chromosomes via orthology "liftover" relative to three different reference assemblies.

M. cinxia	*H. melpomene*	*B. mori*	
15130	15130	15130	total number of protein coding genes in target
14129	14427	14566	number of target genes assigned to chromosome
0.934	0.954	0.963	fraction of target genes assigned to chromosome
454	514	508	number of target scaffolds assigned to chromosomes
6740	8190	7928	number of 1:1 orthologs identified
4607	7150	7534	number of 1:1 orthologs assigned to reference chromosome
0.684	0.873	0.95	fraction of 1:1 orthologs assigned to reference chromosome
