# Supplementary material for: Neo-sex Chromosomes in the Monarch Butterfly, Danaus plexippus
Source: G3 (Bethesda). 2017 Aug 23;7(10):3281–94. doi: 10.1534/g3.117.300187 (PMC5633379; doi:10.1534/g3.117.300187)
Supplement: Supplementary file 4 [file 3281FileS2.pdf]

Supplemental text 1: Identification and analysis of potentially W-linked sequences.

The discovery of a neo-Z chromosome and the cytogenetic analysis in *D. plexippus* suggested the possibility that there may be a neo-W chromosome. Since the genome assembly was generated from a female individual, it is possible that W-linked scaffolds are present in the genome assembly (Zhan *et al.* 2011). Thus, if W-linked scaffolds could be identified in the genome assembly that had obvious homology to neo-Z sequence, it would strongly support the presence of a neo-W chromosome. We thus sought to evaluate whether this might be the case.

The initial effort to identify Z-linked sequences via sequencing coverage analysis applied filters that seemed likely to remove any W-linked scaffolds. Specifically, we excluded short scaffolds (i.e. length < N90) and any scaffolds where samples were missing data (i.e. where median coverage equaled zero for any sample). Since W-linked scaffolds may well be short (given the known complexities of assembling heterochromatic and repetitive sequences) and also have few or no male reads aligning to them, such scaffolds would not have been included in our initial screen for Z-linked scaffolds.

We therefore re-examined the normalized coverage data without a size limit and including all scaffolds, regardless of average coverage values. We identified 12 scaffolds with strong female bias (e.g. more than 2-fold greater average coverage in females than males; Supplemental Text Table 1). Scaffolds below 9kbp were not analyzed further, given their small size, inconsistent coverage patterns, and paucity of genes.

We first attempted to identify any broad-scale patterns of homology between these potentially W-linked scaffolds the Z-linked scaffolds already identified. To do so, we used the PROmer algorithm in MUMmer to query the potentially W-linked scaffolds against a reference of Z-linked scaffolds. No obvious, broad-scale patterns of similarity were detected, as is evident from the PROmer alignments (Supplemental Text Figure 1).

Supplemental Text Table 1. Summary of potentially W-linked scaffolds in the *D. plexippus* genome.

Scaffold	Length	Normalized Coverage						Log2 (Male:Female)	Number of genes
		Female Samples			Male Samples				
		HI035F	M38F	STM123F	STM146M	M36M	HI004M		
DPSCF300409	180121	0.057	0.061	0.067	0	0.073	0	-1.337	7
DPSCF300466	69912	0.342	0.245	0.336	0	0	0	-Inf	3
DPSCF300533	39304	0.285	0.307	0.269	0	0	0	-Inf	1
DPSCF300571	26237	0.114	0.552	0.47	0	0.33	0.231	-1.018	2
DPSCF300619	16724	0.798	0.859	0.47	0.255	0.367	0.231	-1.319	1
DPSCF300683	12133	0.057	0.061	0.067	0	0.073	0	-1.337	1
DPSCF300694	11573	0.57	0.245	0.403	0.255	0.33	0	-1.059	0
DPSCF300753	9117	0.171	0.491	0.47	0	0.147	0.173	-1.823	2
DPSCF300929	5527	0	0	0.873	0	0	0	-Inf	1
DPSCF300955	5344	0.969	0.859	4.702	0.764	0.954	0.635	-1.473	0
DPSCF301041	4863	0.684	0.061	0	0	0.11	0	-2.759	0
DPSCF301071	4728	0.57	1.656	0.403	0.255	0.33	0.289	-1.59	0
DPSCF301073	4725	0	0	0.202	0	0.037	0	-2.457	0
DPSCF301181	4247	0.171	0.061	0.134	0	0	0	-Inf	0

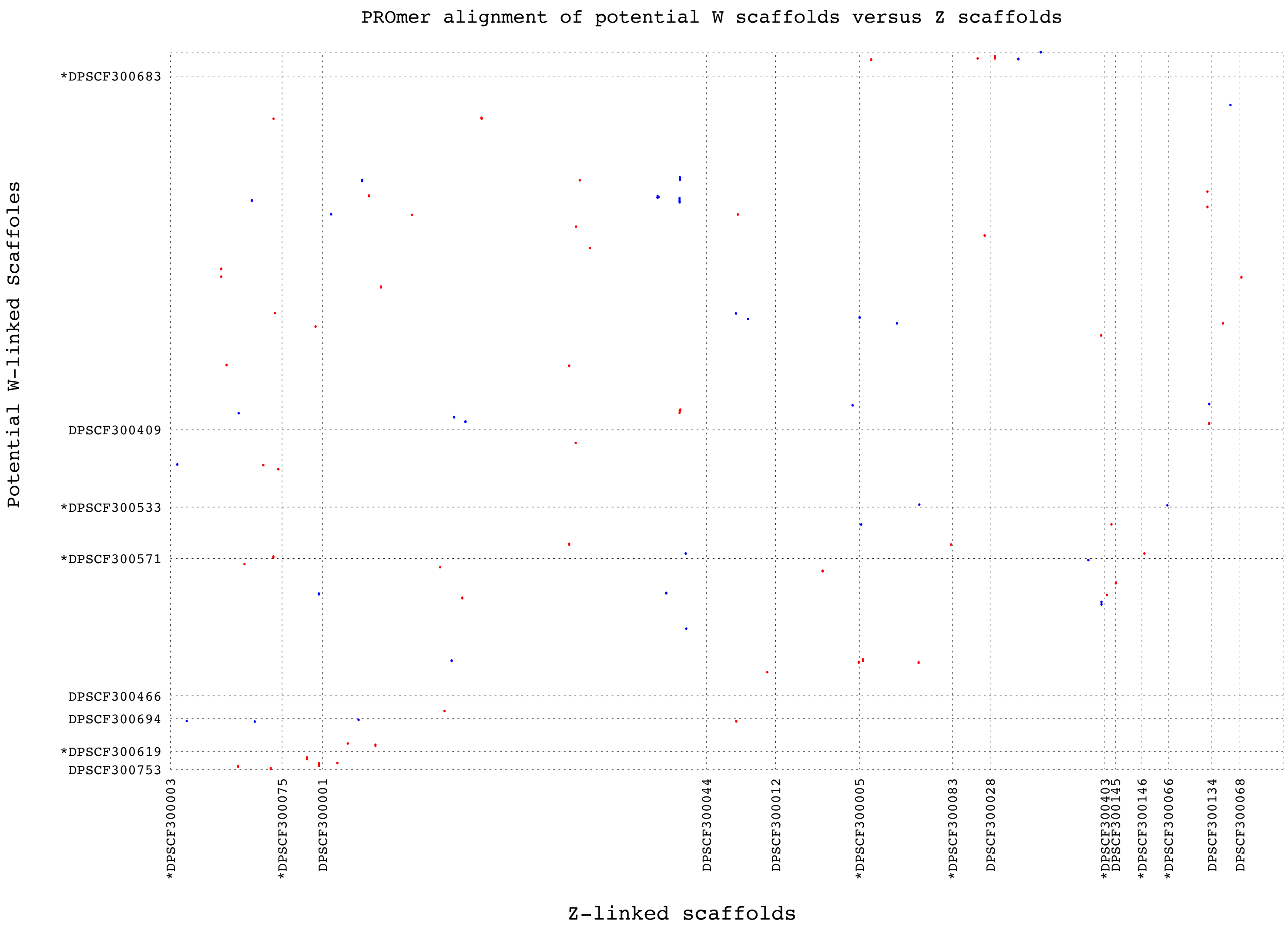

Supplemental Text Figure 1. PROmer alignment of potentially W-linked scaffolds relative to Z-linked reference scaffolds.

Despite this lack of similarity at the level of entire scaffolds, we further investigated the possibility of homology remaining at the level of genes (i.e. that there were gametologs present on the potential W-scaffolds with homology to Z-linked loci). To do so, we performed a tBLASTn search of protein sequences from potential W-linked genes against the entirety of *D. plexippus* genome assembly scaffolds, summarized in Supplemental Text Table 2. Only 6 of the 17 queried proteins had any significant (E-value < 1e-5) similarity to Z-linked scaffolds. However, in each case, the proteins also had at least one and typically multiple hits with much greater significance to autosomal scaffolds. Three of these appear to be related to transposable elements, while the other three may directly function gene regulation or immunity. Overall, these blast results do not suggest any obvious genic homology exists between Z-linked genes and these potentially W-linked sequences.

Supplemental Text Table 2. Summary of BLAST results for potentially W-linked proteins.

**Gene**	**tBLASTn versus *D. plexippus* scaffolds**	**BLASTp versus GenBank non-redundant proteins**
DPOGS200002	Hits multiple scaffolds in the genome, many with much greater significance than any Z scaffolds.	Hits many proteins labeled as "toll precursor".
DPOGS203463	Hits one other autosomal scaffold with greater significance. Only 35 aa long.	No genbank hits
DPOGS203467	Hits multiple scaffolds in the genome, many with much greater significance than any Z scaffolds.	Several hits to Pol-like protein from various inseects; likely a TE.
DPOGS203471	Hits multiple scaffolds in the genome, many with much greater significance than any Z scaffolds.	Homology to proteins predicted to regulate Hox genes (e.g Jim Lovell, TamTrack). Likely has DNA-binding activity, hence the blast hits
DPOGS213849	Hits multiple scaffolds in the genome, many with much greater significance than any Z scaffolds.	Several hits to Pol-like protein from various inseects; likely a TE.
DPOGS213850	Hits multiple scaffolds in the genome, many with much greater significance than any Z scaffolds.	Several hits to Pol-like protein from various inseects; likely a TE.

In conclusion, analysis of sequence homology between Z-linked and potentially W-linked scaffolds in the *D. plexippus* genome assembly does not provide support for the presence of a neo-W. However, these analyses are far from sufficient to exclude the possibility that a neo-W exists. Successful sequencing and assembly of degenerate sex chromosomes like the Y and W is a notoriously difficult task and it is a distinct possibility that much W-sequence (neo or otherwise) is not represented in the current genome assembly. Thus, it is likely that much further focused effort and novel data will be required to robustly assess the possibility that *D. plexippus* harbors a neo-W chromosome.

The identification here of several potentially W-linked sequences harboring with functional protein-coding genes is noteworthy, given the paucity of known W-linked protein-coding loci in Lepidoptera (Sahara *et al.* 2011; Van't Hof *et al.* 2013). This presents an interesting opportunity for future study, but given the lack of homology to the Z, it is not of immediate consequence to the question of a neo-W in *D. plexippus*.

Works Cited

Sahara K., Yoshido A., Traut W., 2011 Sex chromosome evolution in moths and butterflies. Chromosome Res **20**: 83–94.

Van't Hof A. E., Nguyen P., Dalíková M., Edmonds N., Marec F., Saccheri I. J., 2013 Linkage map of the peppered moth, Biston betularia (Lepidoptera, Geometridae): a model of industrial melanism. Heredity **110**: 283–295.

Zhan S., Merlin C., Boore J. L., Reppert S. M., 2011 The Monarch Butterfly Genome Yields Insights into Long-Distance Migration. Cell **147**: 1171–1185.
